# Geographical Representativeness of Published and Ongoing Randomized Controlled Trials. The Example of: Tobacco Consumption and HIV Infection

**DOI:** 10.1371/journal.pone.0016878

**Published:** 2011-02-09

**Authors:** Nizar Ahmad, Isabelle Boutron, Agnes Dechartres, Pierre Durieux, Philippe Ravaud

**Affiliations:** 1 INSERM, U738, Paris, France; 2 AP-HP (Assistance Publique des Hôpitaux de Paris), Hôpital Hôtel Dieu, Centre d'Epidémiologie Clinique, Paris, France; 3 Université Paris Descartes, Faculté de Médecine, Paris, France; 4 Centre de Médecine Fondée sur les Preuves (EHESP, HAS, INSERM, APHP), Paris, France; 5 Santé Publique et Informatique Médicale, Faculté de Médecine, Université Paris Descartes, Paris, France; Institut National de la Santé et de la Recherche Médicale, France

## Abstract

**Background:**

The challenge for evidence-based healthcare is to reduce mortality and the burden of diseases. This study aimed to compare where research is conducted to where research is needed for 2 public health priorities: tobacco consumption and HIV infection.

**Methods:**

We identified randomized controlled trials (RCTs) included in Cochrane systematic reviews published between 1997 and 2007 and registered ongoing RCTs identified in January 2009 through the World Health Organization's International Clinical Trials Registry Platform (WHO-ICTRP) evaluating interventions aimed at reducing or stopping tobacco use and treating or preventing HIV infection. We used the WHO and World Bank reports to classify the countries by income level, as well as map the global burden of disease and mortality attributable to tobacco use and HIV infection to the countries where the trials performed.

**Results:**

We evaluated 740 RCTs included in systematic reviews and 346 ongoing RCTs. For tobacco use, 4% of RCTs included in systematic reviews and 2% of ongoing trials were performed in low- and middle-income countries, even though these countries represented 70% of the mortality related to tobacco use. For HIV infection, 31% of RCTs included in systematic reviews and 33% of ongoing trials were performed in low- and middle-income countries, even though these countries represented 99% of the mortality related to HIV infection.

**Conclusions:**

Our results highlight an important underrepresentation of low- and middle-income countries in currently available evidence (RCTs included in systematic reviews) and awaiting evidence (registered ongoing RCTs) for reducing or stopping tobacco use and treating or preventing HIV infection.

## Introduction

The main challenge for public health policies is to reduce mortality and the burden of disease. This aim implies having adequate evidence to guide health care providers and policymakers for implementing the most efficient and cost-effective interventions. More than two-thirds of the world's population live in low- and middle-income countries [1], [2], and 93% of the burden of preventable mortality occurs in these countries [3]. The shortage of resources in low- and middle-income countries paradoxically increases the need for reliable healthcare evidence to prioritize the use of these scarce resources [4], [5]. Such evidence is essential to determine the prevention and therapeutic strategies that work best but also under which circumstances they work and how best to deliver them [6].

In this context, it could be useful to tailor research to the needs of the particular population and to the context (socio-cultural, access to services, etc.). Addressing context-specific questions is fundamental to designing interventions that improve health [7], [8]. Results of research performed in high-income countries cannot be easily transposed to low- and middle-income countries. The extent to which questions addressed by researchers in high-income countries are relevant to patients and physicians in low-income countries is largely unknown.

This study aimed to compare where research is conducted to where research is needed according to the attributable burden of disease, mortality and prevalence. We compared high-income countries to low- or middle-income countries defined by the 2008 World Bank classification. We considered both current available evidence (i.e., randomized controlled trials [RCTs] included in systematic reviews) and awaiting evidence (i.e., RCTs registered on the World Health Organization's International Clinical Trials Registry Platform [WHO-ICTRP]) that provide data for current clinical decision making and future public health policies. We focused on 2 public-health priorities, tobacco use and HIV infection, because they are ranked among the top 5 leading causes of mortality in the world [9].

## Methods

### I. Classification of countries

We classified the countries according to the 2008 World Bank classification that divides the world into income categories (low, middle, and high) according to economies in terms of gross region national income per capita and in geographic regions for low- and middle-income economies only. We considered trials performed in high-income countries (country with a per capita income of ≥$11 906 in 2008 [e.g., USA, European Union [EU], Canada, Australia and other]) as one group and trials performed in low- and middle-income countries as another group [9], [10]. In addition, we grouped low- and middle-income countries into 6 geographic regions according to the 2008 World Bank classification (i.e., East Asia and Pacific, Eastern Europe and Central Asia, Latin America and Caribbean, Middle East and North Africa, South Asia, Sub-Saharan Africa).

### II. Where research is conducted

To describe the context (i.e., geographic region and socioeconomic situation of the countries where the trial took place) of research performed in these fields, we focused on the context of currently available evidence and awaiting evidence that could be used for clinical decision making. Because RCTs are the gold standard to assess the effectiveness of interventions, we evaluated the context of all RCTs included in Cochrane systematic reviews (i.e., context of current evidence) and all ongoing RCTs in the registries of the WHO-ICTRP (i.e., context of awaiting evidence). We focused on systematic reviews performed by the Cochrane collaboration because this international, not-for-profit organisation is a source of high-quality, reliable health information providing up-to-date knowledge about the effects of health care. Further, an important goal of the Cochrane collaboration is the dissemination of information to low- and middle-income countries. The Cochrane Library is freely available to all residents of low-income countries.

#### a. Identification of RCTs included in Cochrane systematic reviews

We identified all systematic reviews of interventions aimed at reducing or stopping tobacco use and those aimed at treating or preventing HIV infection published in the Cochrane database of systematic reviews between January 1997 and December 2007. We searched for the following terms in the title, abstract or the MeSH terms: “smoking cessation” OR “tobacco use cessation” OR “smoking reduction” OR “tobacco reduction” OR “smoking abstinence” OR “tobacco abstinence” for tobacco use and “HIV” OR “human immunodeficiency virus” OR “AIDS” OR “acquired immunodeficiency syndrome” OR “sexually transmitted diseases” for HIV infection. Titles and abstracts were then screened by one of us (NA) to identify the relevant systematic reviews. The full texts for selected abstracts were retrieved and reviewed by one of us (NA) to determine the eligibility of systematic reviews for inclusion. Another reviewer (IB) checked the adequate selection of the abstracts and confirmed the adequate exclusion of full-text articles.

Reports were included if the study was identified as a systematic review or meta-analysis of interventions aimed at reducing tobacco consumption or preventing or treating HIV infection. We excluded systematic reviews focusing on a specific setting, except when this setting concerned developing countries (e.g., intervention for tobacco cessation in a dental setting) or a specific population (e.g., tobacco cessation for hospitalised patients). Excluding these systematic reviews allowed for a relatively homogeneous sample of systematic reviews aimed at providing conclusions on treatment effect whatever the context. We also excluded systematic reviews of prevention or treatment of complications of HIV infection (e.g., opportunistic infections, Kaposi's sarcoma) and those evaluating an intervention for another disease among subjects infected with HIV (e.g., treatment of anaemia or tuberculosis infection in people with HIV infection).

#### b. Identification of ongoing RCTs

In January 2009, we searched the WHO-ICTRP (http://www.who.int/trialsearch/) for all ongoing RCTs registered in the platform's 10 clinical trials registries: Australian New Zealand Clinical Trials Registry (ANZCTR), Chinese Clinical Trial Register (ChiCTR), ClinicalTrials.gov, Clinical Trials Registry - India (CTRI), German Clinical Trials Register (DRKS), Iranian Registry of Clinical Trials (IRCT), International Standard Randomised Controlled Trials Number (ISRCTN.org), Japan Primary Registries Network, The Netherlands National Trial Register (NTR), and Sri Lanka Clinical Trials Registry (SLCTR). We used this platform because it allows access to all primary registries meeting the WHO criteria and the requirements of the International Committee of Medical Journal Editors [11].

We searched for RCTs in the “conditions” menu of the platform using the topics “smoking” for tobacco use and “HIV infection” for HIV infection and in the “recruitment” menu of the “advanced search” feature, using “recruiting” to select only ongoing RCTs.

#### c. Data collection

One of us (NA) screened all records obtained by this search to select the relevant RCTs (i.e., all RCTs of interventions aimed at reducing or stopping tobacco consumption or preventing or treating HIV infection).

For RCTs included in systematic reviews, we collected data on the individual RCTs included in the review. To avoid collecting data several times from an RCT included in several systematic reviews, we recorded all RCTs in an EndNote data file (EndNote for Windows Version X2, Bld (3210)). We systematically searched this file for reports with the same authors and excluded duplicates.

If the country where the trial was performed was not mentioned in the systematic review, we retrieved the published report for the trial to obtain more information.

As a quality assurance exercise, another author (AD) collected data on a random sample of 102 RCTs included in systematic reviews.

For ongoing trials, we collected data from the trial records available in the WHO-ICTRP and in the primary registry's records.

For each RCT included in a systematic review or registered, we checked whether the trial was performed in a high-income or a low- or middle-income country. We considered trials performed in both high-income and low- and middle-income countries as trials of low- and middle-income countries.

We also collected data on the intervention (pharmacological or nonpharmacologic treatment) and the number of participating countries.

### III. Where research is needed

The following indicators were elaborated:

#### 1. Prevalence of tobacco use and HIV infection

To obtain the prevalence of tobacco use and HIV infection in the world, in high-income countries and in different regions of low- and middle-income countries, we used the World Bank reports for number of smokers [12] and WHO reports for number of people living with HIV infection [13], [14].

#### 2. Mortality and global burden of disease (GBD)

The global burden of disease (GBD) reflects the burden of disease by disability-adjusted life years (DALYs). This time-based measure combines years of life lost due to premature mortality and that lost due to time lived in states of less than full health. The DALY measure was developed in the original GBD 1990 study to assess the burden of disease consistently across diseases, risk factors and regions [15].

We used the data (WHO assessment of the GBD for 2000–2002 compiled and updated by the World Bank) related to the GBD (DALYs) and mortality attributable to tobacco use and HIV infection in the world, in high-income countries and in low- and middle-income countries [16].

### IV. Geographic representation

For an adequate representation of the contrast between the area where trials are needed and the area where trials are conducted, we constructed an area cartogram according to the method developed by Gastner and colleagues [17]. Area cartograms are maps in which the sizes of geographic regions such as countries are not proportional to the area on the ground but, rather, appear in proportion to their population or some other demographic feature such as disease prevalence. We used such maps to compare the area for number of people smoking and with HIV infection to where RCTs have been and are being conducted.

### Statistical analysis

We used descriptive statistics; categorical variables are described with frequencies and percentages. All data analyses involved use of SAS for Windows, Release 9.1 (SAS Inst., Cary, NC).

Cartographic representation was developed with use of Migratio 8.0 (Stéphane Le Rouzic, Migratio.fr, Rennes, France) and ScapeToad cartography software (Chôros Laboratory, EPFL-ENAC-INTER, Lausanne, Switzerland).

## Results

### Where research is conducted

**Figure 1:**
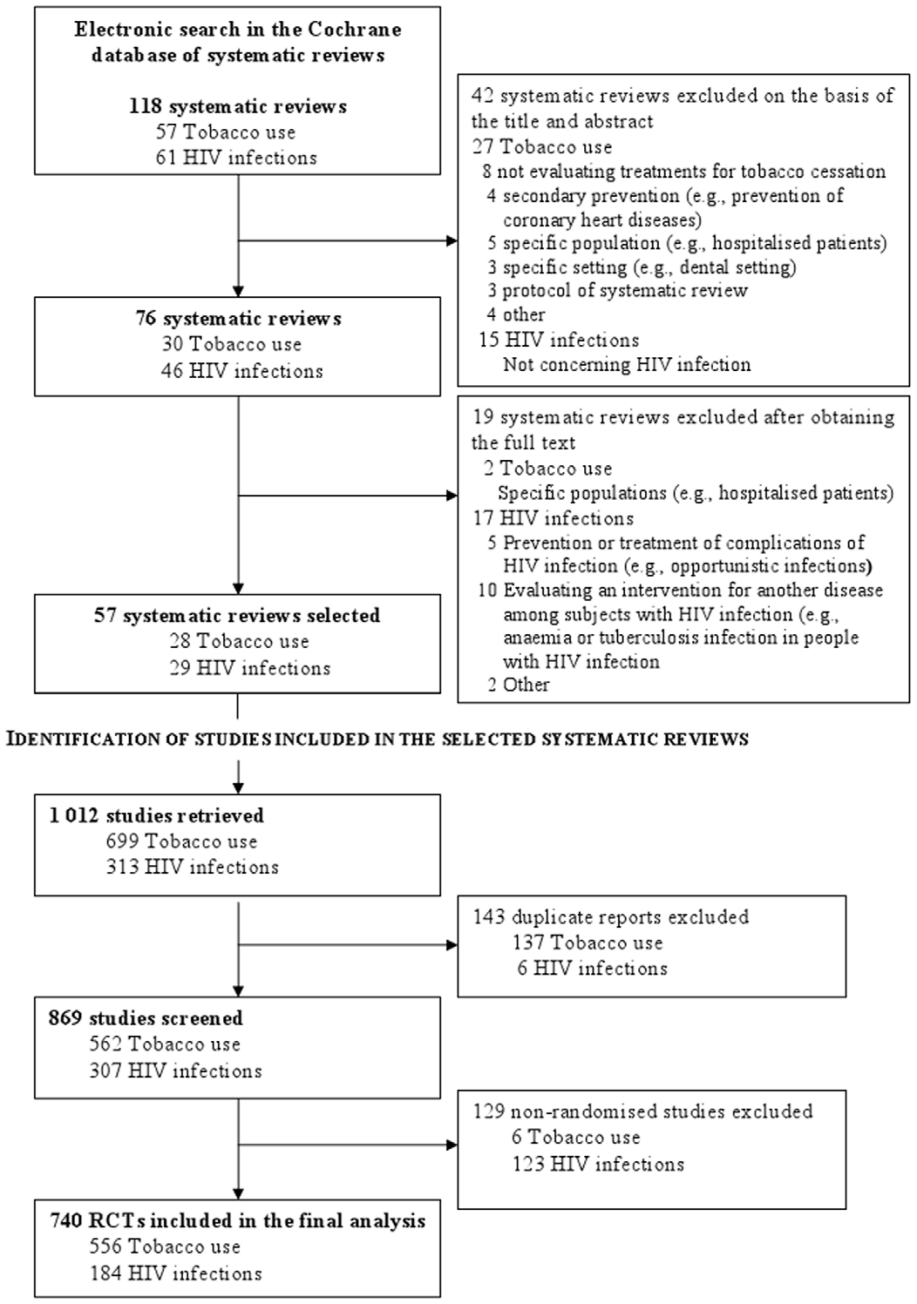
Flow diagram of Cochrane systematic reviews selected in the study and retrieval of randomized controlled trials (RCTs) from these reviews.

**Figure 2:**
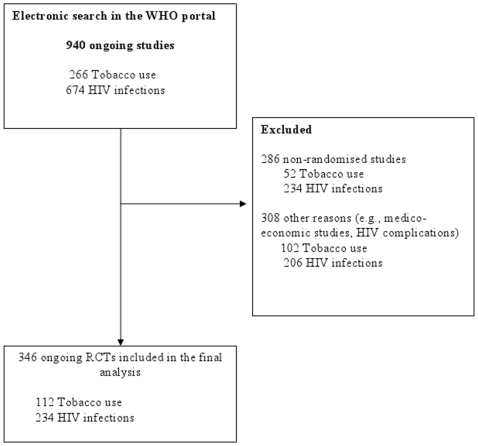
Flow diagram of reports of ongoing RCTs selected from International Clinical Trial Registries* via the World Health Organization (WHO) portal. * International clinical trial registries: Clinicaltrials.gov, Australian New Zealand Clinical Trials Registry, Chinese Clinical Trial Register, Clinical Trials Registry - India, German Clinical Trials Register, Iranian Registry of Clinical Trials, Japan Primary Registries Network, International Standard Randomised Controlled Trials Number, The Netherlands National Trial Register, Sri Lanka Clinical Trials Registry.

The flow of RCTs included in the selected systematic reviews and ongoing RCTs through the study is in Figures 1 and 2, respectively. The electronic search yielded 118 systematic reviews, of which 57 (28 of tobacco use, 29 of HIV infection) were selected. From these systematic reviews, data for 1 012 trials were retrieved; 143 duplicate reports and reports of 129 nonrandomised trials were excluded. We included in the final analysis data for 740 RCTs (556 of tobacco use, 184 of HIV infection). Of the 940 ongoing RCTs (266 of tobacco use, 674 of HIV infection) identified in the international clinical trial registries, we included in the final analysis data for 346 (112 of tobacco use, 234 of HIV infection).

The characteristics of the RCTs and ongoing RCTs are in Table 1.

**Table 1:** Characteristics of RCTs of tobacco use and HIV infection included in selected Cochrane systematic reviews and ongoing RCTs selected from international clinical trial registries.

	Total				Tobacco use			HIV infection		
	All		RCTs	Ongoing RCTs	All	RCTs	Ongoing RCTs	All	RCTs	Ongoing RCTs
	n = 1 086 (%)		n = 740 (%)	n = 346 (%)	n = 668 (%)	n = 556 (%)	n = 112 (%)	n = 418 (%)	n = 184 (%)	n = 234 (%)
**Intervention**										
• PT	489 (45.0)		283 (38.2)	206 (59.5)	252 (37.7)	201 (36.2)	51 (45.5)	237 (56.7)	82 (44.6)	155 (66.2)
• NPT	597 (55.0)		457 (61.8)	140 (40.5)	416 (62.3)	355 (63.8)	61 (54.5)	181 (43.3)	102 (55.4)	79 (33.8)
									
**Number of participating countries**										
• 1		1 004 (92.5)	695 (94.0)	309 (89.3)	639 (95.7)	529 (95.1)	110 (98.2)	365 (87.3)	166 (90.2)	199 (85.0)
• 1–5		38 (3.5)	15 (2.0)	23 (6.7)	8 (1.2)	6 (1.1)	2 (1.8)	30 (7.2)	9 (4.9)	21 (9.0)
• 6–10		10 (0.9)	4 (0.5)	6 (1.7)	4 (0.6)	4 (0.7)	0	6 (1.4)	0	6 (2.6)
• >10		12 (1.1)	4 (0.5)	8 (2.3)	2 (0.3)	2 (0.4)	0	10 (2.4)	2 (1.1)	8 (3.4)
• Not reported		22 (2.0)	22 (3.0)	--	15 (2.2)	15 (2.7)	--	7 (1.7)	7 (3.8)	--

RCTs: randomized controlled trials. PT: pharmacologic intervention. NPT: nonpharmacologic intervention.

### Geographic representation

#### Tobacco use trials

**Figure 3:**
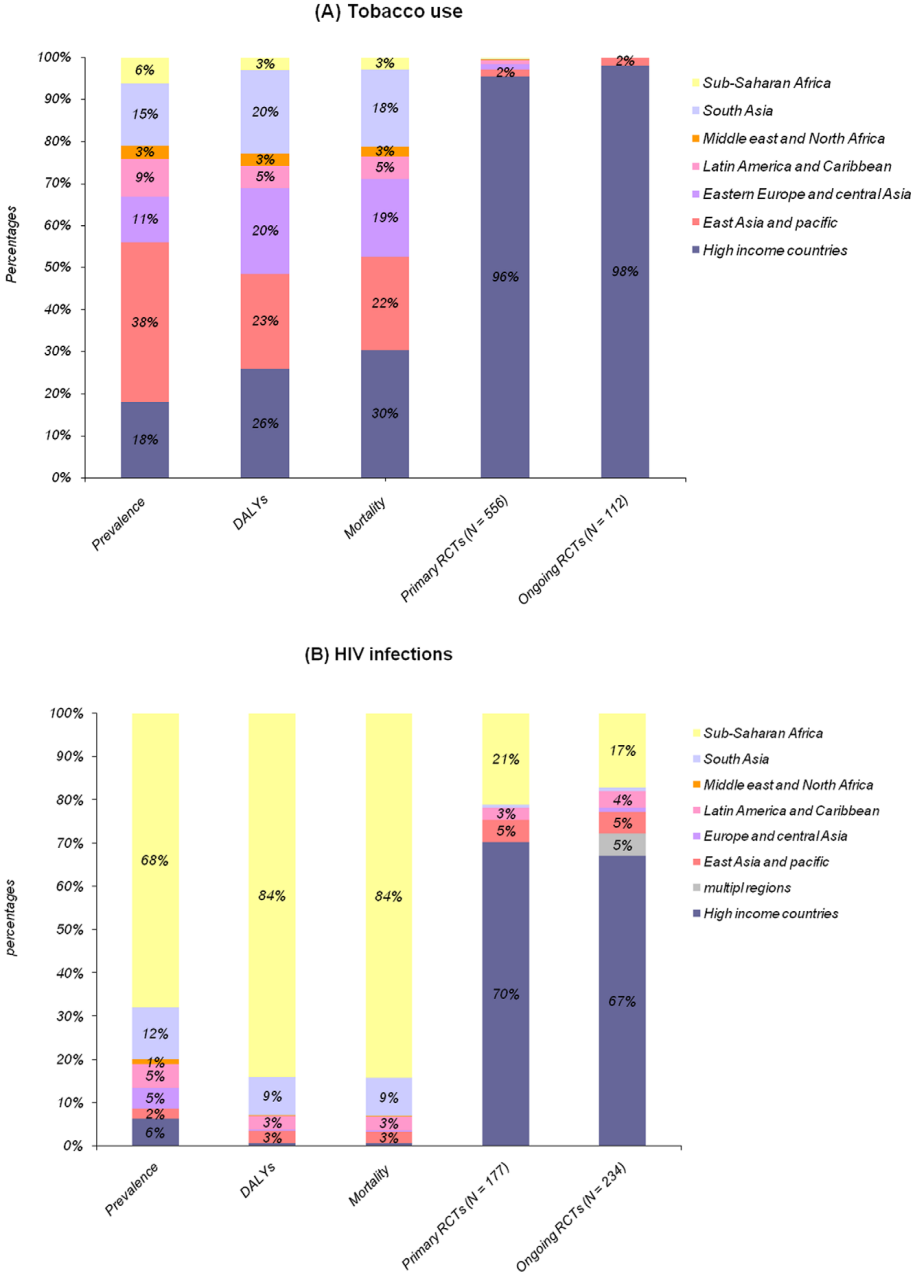
Prevalence, burden of disease (in disability-adjusted life-years [DALYs]), attributable mortality and percentage of RCTs included in Cochrane systematic reviews and ongoing RCTs selected from international clinical trial registries for different regions of the world for trials of tobacco use (A) and HIV infection (B).

**Figure 4:**
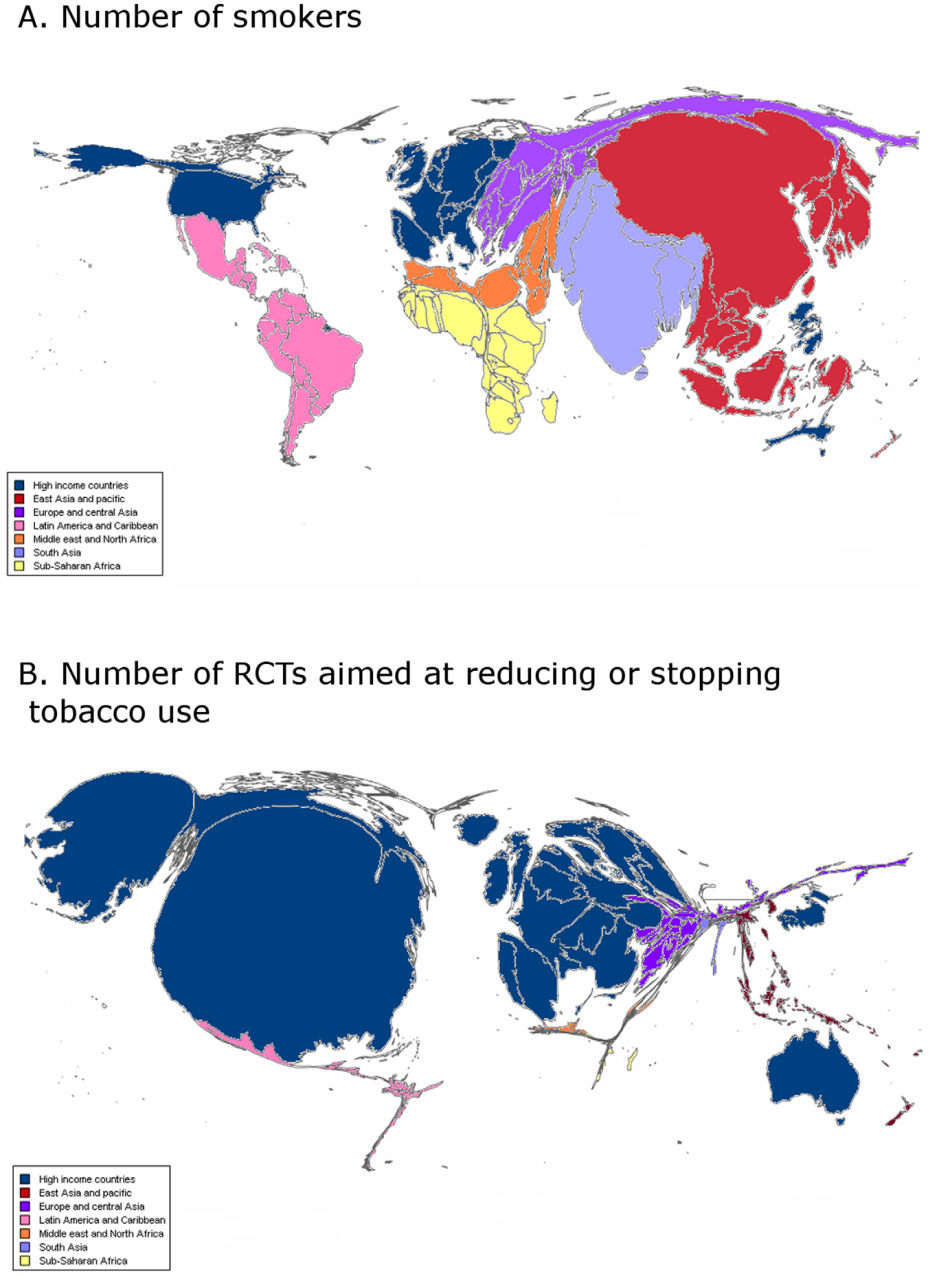
Area cartograms showing the sizes of countries in proportion to A) the number of smokers* and B) the number of RCTs (RCTs included in Cochrane systematic reviews and ongoing RCTs) aimed at reducing or stopping tobacco use^†^. * Source: World Bank's 2005 World Development Indicators, 2005. Table 2.18 Health: risk factors and future challenges. The World Bank sourced this in turn from the World Health Organization's 2004 World Health Report, Tobacco Control Country Profiles 2003. ^†^ The number of RCTs (primary and ongoing) was classified by main regions according to the 2008 World Bank classification: high-income countries, East Asia and Pacific, Eastern Europe and Central Asia, Latin America and Caribbean, Middle East and North Africa, South Asia, Sub-Saharan Africa. For the construction of the map we divided the number of trials performed in each region by the number of countries in each region.

The geographic comparison of location of RCTs of tobacco use and mortality and GBD (in DALYs) attributable to tobacco use is in Table 2 and Figure 3A. In total, 96% of RCTs included in systematic reviews and 98% of ongoing RCTs were performed in high-income countries. Both kinds of trials were performed mainly in the USA (314 [57%] and 68 [61%], respectively) and the EU (144 [26%] and 27 [24%], respectively). Only 24 (4%) RCTs included in systematic reviews and 2 (2%) ongoing RCTs were carried out in low- or middle-income countries, although these countries represented 74% of the GBD (in DALYs) and 70% of the mortality attributable to tobacco use and 82% of the smokers. Only 11 of 341 (3%) trials and 2 of 61 (3%) ongoing RCTs assessing nonpharmacologic treatment were performed in low- and middle-income countries. The highest prevalence of tobacco use concerns East Asia and Pacific countries (38%). This area represents 22% of the mortality attributable to tobacco use. In this area, only 9 RCTs were performed, and only 2 registered RCTs are ongoing. Similarly, no ongoing trial is registered in Eastern Europe and Central Asia, South Asia, Latin America and Caribbean, the Middle East and North Africa, and Sub-Saharan Africa, which overall represent nearly half of the mortality attributable to tobacco use. Figure 4 highlights the differences in geographic representation between regions with a high prevalence of smokers and where trials are conducted.

**Table 2:** Tobacco use: smoking prevalence, burden of disease (in disability-adjusted life-years [DALYs]), attributable mortality, RCTs included in Cochrane systematic reviews and ongoing RCTs selected from international clinical trial registries for high-income and low- and middle-income countries.

Countries	Attributable DALYs (thousands)	Attributablemortality (thousands)	Smoking prevalence (millions)	Total RCTs	PT RCTs	NPT RCTs	Total ongoing RCTs	PT ongoing RCTs	NPT ongoing RCTs
	n = 72 919 (%)	n = 4 802 (%)	n = 11 220 (%)	n = 541 (%)	n = 200 (%)	n = 341 (%)	n = 112(%)	n = 51 (%)	n = 61(%)
**High-income countries**	18 900 (25.9)	1 462 (30.4)	202 (18.0)	517 (95.6)	187 (93.5)	330 (96.8)	110 (98.2)	51 (100)	59 (96.7)
**Low- or middle-income countries**	54 019 (74.1)	3 340 (69.6)	920 (82.0)	24 (4.4)	13 (6.5)	11 (3.2)	2 (1.8)	0	2 (3.3)
• East Asia and Pacific	16 518 (22.7)	1 059 (22.1)	429 (38.0)	9 (1.6)	4 (2.0)	5 (1.5)	2 (1.8)	0	2 (3.3)
• Eastern Europe and central Asia	14 769 (20.3)	897 (18.7)	122 (11.0)	7 (1.3)	4 (2.0)	3 (0.9)	0	0	0
• South Asia	14 452 (19.8)	879 (18.3)	178 (15.0)	1 (0.2)	0	1 (0.3)	0	0	0
• Latin America and Caribbean	3 957 (5.4)	250 (5.2)	98 (9.0)	5 (0.9)	4 (2.0)	1 (0.3)	0	0	0
• Middle east and North Africa	2 153 (2.9)	121 (2.5)	37 (3.0)	1 (0.2)	1 (0.5)	0	0	0	0
• Sub-Saharan Africa	2 171 (3.0)			1 (0.2)	0	1 (0.3)	0	0	0

RCTs: randomized controlled trials. PT: pharmacologic intervention. NPT: nonpharmacologic intervention. GBD: global burden of disease.

#### HIV infection trials

The geographical comparison of location of RCTs and mortality and GBD (in DALYs) attributable to HIV infection is in Table 3 and Figure 3B. In total, 70% of RCTs included in systematic reviews and 67% of ongoing RCTs were performed in high-income countries. Only 54 (31%) RCTs and 77 (33%) ongoing RCTs were carried out in low- or middle-income countries, although these countries represented 99% of the GBD, 99% of the mortality and 94% of the people with HIV infection. About 16% (16/99) of trials and 26% (20/78) of ongoing RCTs assessing nonpharmacologic treatments were performed in low- and middle-income countries.

**Table 3:** HIV infection: AIDS prevalence, burden of disease (in disability-adjusted life-years [DALYs]), attributable mortality, RCTs included in Cochrane systematic reviews and ongoing RCTs selected from International Clinical Trials Registries for high-income and low- and middle-income countries.

Countries	Attributable DALYs (thousands)	Attributable mortality (thousands)	HIV infection prevalence(thousands)	Total RCTs	PT RCTs	NPT RCTs	Total ongoing RCTs	PT ongoing RCTs	NPT ongoing RCTs
	n = 67 568 (%)	n = 2 440 (%)	n = 33 200(%)	n = 177(%)	n = 78(%)	n = 99(%)	n = 234(%)	n = 156(%)	n = 78(%)
**High-income countries**	445 (0.6)	15 (0.6)	2 100 (6.3)	123 (69.5)	40 (51.3)	83 (83.8)	157 (67.1)	99 (63.5)	58 (74.3)
**Low- or middle-income countries**	67 141 (99.4)	2 425 (99.4)	31 100 (93.7)	54 (30.5)	38 (48.7)	16 (16.2)	77 (32.9)	57 (36.5)	20 (25.7)
• Sub-Saharan Africa	56 795 (84.1)	2 057 (84.3)	22 500 (67.9)	37 (20.9)	29 (37.1)	10 (10.1)	40 (17.1)	28 (17.9)	12 (15.3)
• South Asia	5 846 (8.7)	214 (8.8)	4 000 (12.0)	1 (0.6)	0	0	2 (0.9)	0	2 (2.6)
• Latin America and Caribbean	2 099 (3.1)	74 (3.1)	1 800 (5.4)	5 (2.8)	2 (2.6)	2 (2.0)	9 (3.8)	7 (4.5)	2 (2.6)
• East Asia and pacific	2 026 (2.9)	68 (2.8)	800 (2.4)	9 (5.1)	5 (6.4)	4 (4.1)	12 (5.1)	10 (6.4)	2 (2.6)
• Eastern Europe and central Asia	277 (0.4)	8 (0.3)	1 600 (4.8)	0	0	0	2 (0.9)	0	2 (2.6)
• Middle east and North Africa	99 (0.2)	--	--	0	0	0	0	0	0
• Multiple regions	--			2 (1.1)	2 (2.6)	--	12 (5.1)	12 (7.7)	--

RCTs: randomized controlled trials. PT: pharmacologic intervention. NPT: nonpharmacologic intervention. GBD: global burden of disease.

**Figure 5:**
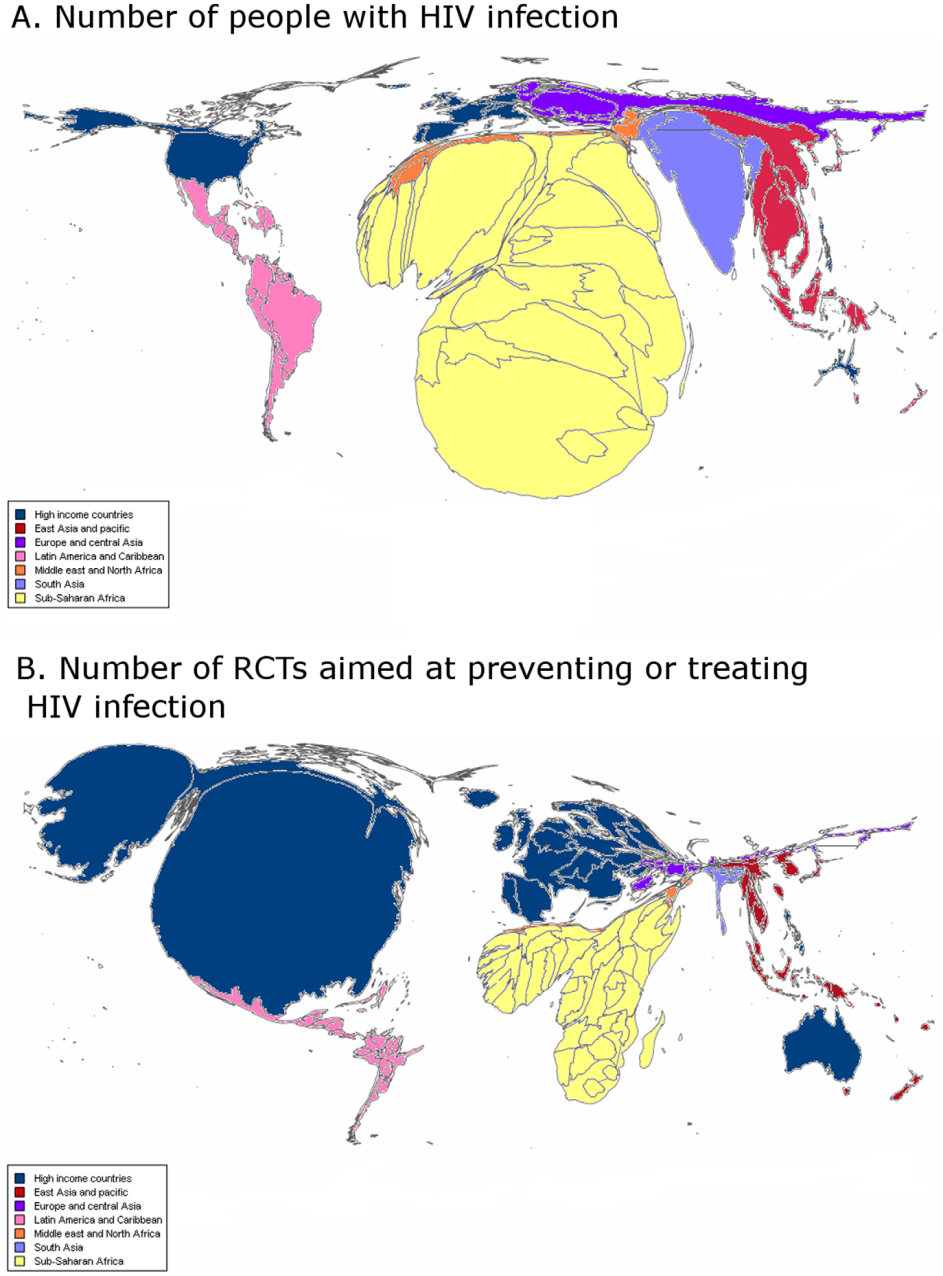
Area cartograms showing the sizes of countries in proportion to A) the number of people with HIV infection* and B) the number of RCTs (primary and ongoing RCTs) aimed at treating or preventing HIV infection^†^. *Source: report on the global AIDS epidemic, UNAIDS/WHO, July 2008. ^†^The number of RCTs (primary and ongoing) was classified by main regions according to the 2008 World Bank classification: high-income countries, East Asia and Pacific, Eastern Europe and Central Asia, Latin America and Caribbean, Middle East and North Africa, South Asia, Sub-Saharan Africa. For the construction of the map we divided the number of trials performed in each region by the number of countries in each region.

Only 37 (21%) RCTs and 40 (17%) ongoing RCTs were performed in Sub-Saharan Africa, which represents 84% of the GBD and 84% of the mortality attributable to HIV infection and 68% of the people with HIV infection. Similarly, only 2 ongoing RCTs are being performed in South Asia, although this area represents 9% of the GBD, 9% of the mortality and 12% of the people with HIV infection. Figure 5 highlights the differences in geographic representation between the regions with a high prevalence of people with HIV infection and where trials are conducted.

## Discussion

This study evaluated the context of clinical research (i.e., where research is performed) for 2 public health priorities: tobacco use and HIV infection. We described the location of 740 RCTs included in Cochrane systematic reviews and 346 ongoing RCTs identified through international clinical trial registries. Our results highlight a gap between where trials are conducted and where research is needed in terms of GBD, mortality and prevalence of tobacco use and HIV infection. Most ongoing and RCTs were performed in high-income countries, even though most of the mortality and GBD concerns low- and middle-income countries. Only 4% and 32% of the total and ongoing RCTs performed in the fields of tobacco use and HIV infection, respectively, were conducted in low- and middle-income countries. However, low- and middle-income countries represent 70% and 99% of the mortality attributable to tobacco use and HIV infection, respectively [13], [18].

The number of trials of tobacco use performed in low- and middle-income countries was particularly low (4%) as compared with trials of HIV infection performed in these countries (about one-third).

Previous studies have highlighted the underrepresentation of research addressing priority issues for low- and middle-income countries [19]. Some authors highlighted the poor association of GBD and reports of RCTs published in high-impact-factor journals [19], [20]. Swingler et al. [21] analysed nearly 3 000 systematic reviews and assessed the correlation between the number of systematic reviews undertaken and the burden of disease: only a few systematic reviews focused on some diseases affecting a large number of the world's population. Sheriff showed that only 3% of mental disease research is performed in low- and middle-income countries [22], but the religious and cultural individuality of these countries could greatly hamper implementation of any mental health interventions recommended by research performed in high-income countries. These low research percentages are probably related to the limited financial investment in research for low- and middle-income countries because of the phenomenon of the “10/90 gap”. The Global Forum has highlighted that of the US$73 billion invested annually in global health research, less than 10% is spent on research into the health problems that account for 90% of the GBD [4], [23], [24]. This gap is also reflected in the low proportion of publications from research performed in low- and middle-income countries [25].

Contrary to previous research, we focused on 2 public health priorities (tobacco use, responsible for non-communicable chronic diseases, and HIV infection, a communicable chronic disease) relevant for high-, low- and middle-income countries. Our study is the first to focus on existing evidence (trials included in systematic reviews) and on awaiting evidence that will guide future evidence-based practices.

With the evidence of the effectiveness of an intervention, some might assume that the challenge is to make the intervention available in low- and middle-income countries [26]. However, the results of trials performed in high-income countries may not be easily applied to low- and middle-income countries. Many interventions shown to be efficacious in high-income countries are not similarly effective when carried out in other contexts [26], [27]. The populations can differ, with people consulting late, frequently using self-medication, unable or unwilling to adhere to treatment, and having several co-morbidities (malnourishment, anaemia, malaria, etc.), in addition to the behavioural and cultural differences [28], [29]. Further, the contextual factors differ greatly, particularly the social and cultural context, as well as the available facilities and infrastructures. The lack of studies performed in a relevant location is particularly problematic for nonpharmacologic interventions targeting behavioural changes (e.g., education, counselling), because the results of such trials could be strongly influenced by cultural conditions [20], [30]. The influence of the cultural context is important, even between different high-income countries [31].

Recently, the Global Alliance for Chronic Disease (GACD) was created to address this imbalance issue [32]. The GACD brings together 6 major national health research councils representing 80% of all public research funding in the world to coordinate research activities that address the prevention and treatment of chronic disease on a global scale. One essential goal of the GACD is to focus on chronic disease in low- and middle-income countries and to coordinate research into low-cost interventions. This initiative should be important in limiting the worldwide imbalance in health research resources [7].

Similarly, the High Level Taskforce on Innovative International Financing for Health Systems was created in September 2008 to help fill national financing gaps to reach the health Millennium Development Goals in low-income countries. One key challenge is to obtain evidence for what works according to different settings [33].

Our study has some limitations. First, we focused on only 2 medical areas, and these results should be confirmed in other medical areas. However, we chose tobacco use and HIV infection because they are among the first 5 causes of mortality in the world [9]. Second, we selected only RCTs included in Cochrane systematic reviews because these systematic reviews are known to be of high quality, and we did not consider non-Cochrane systematic reviews [34], [35], [36], [37], [38]. As well, the Cochrane collaboration seeks to actively encourage the participation of reviewers from developing countries and to prioritize reviews focusing on determinants of health that are particularly pertinent to low- and middle-income countries [1], [4]. Finally, our results may underestimate the number of ongoing RCTs performed in low- and middle-income countries because we focused on only available data (i.e., trials that were registered in a trial registry available on the WHO portal). We cannot exclude that some trials performed in low-income countries were not registered.

In conclusion, clinical research into tobacco use and HIV infection is not performed in the world locations most affected in terms of GBD and mortality because we found important underrepresentation of such research in low- and middle-income countries. The GBD measured by mortality or DALYs could be a useful aid in deciding where to conduct health research. The measure should help in conducting RCTs in an appropriate context to improve clinical research practice and decrease the GBD [39].
